# Unusual Onset of Celiac Disease and Addison’s Disease in a 12-Year-Old Boy

**DOI:** 10.3390/ijerph14080855

**Published:** 2017-07-29

**Authors:** Francesco Miconi, Emanuela Savarese, Giovanni Miconi, Gabriele Cabiati, Valentina Rapaccini, Nicola Principi, Susanna Esposito

**Affiliations:** 1 Paediatric Clinic, Azienda Ospedaliera di Terni, 05100 Terni, Italy; francesco.miconi90@gmail.com (F.M.); manu-s84@hotmail.it (E.S.); g.miconi@aospterni.it (G.M.); gabrielecabiati@libero.it (G.C.); rapaccinivalentina@gmail.com (V.R.); 2 Pediatric Highly Intensive Care Unit, Fondazione IRCCS Ca’ Granda Ospedale Maggiore Policlinico, Università degli Studi di Milano, 20122 Milan, Italy; nicola.principi@unimi.it; 3 Paediatric Clinic, Department of Surgical and Biomedical Sciences, Università degli Studi di Perugia, 06123 Perugia, Italy

**Keywords:** Addison’s disease, autoimmune diseases, celiac disease, gluten-free diet

## Abstract

Background: Celiac disease (CD) is an autoimmune disorder deriving from an aberrant adaptive immune response against gluten-containing grains in genetically predisposed subjects. In a number of patients, CD is associated with one or more other autoimmune diseases. Primary Addison’s disease (AD) and CD may co-exist, although this association is relatively uncommon in children. In addition, it is not precisely defined whether a gluten-free diet influences the course of AD. Case presentation: A case of CD in a 12-year-old boy presenting as acute adrenal insufficiency is described here. A gluten-free diet had a significant therapeutic role in this case, wherein most of the clinical signs and symptoms of AD disappeared in a few days. In addition, the dosage of cortisol acetate, initially administered to treat the AD, was able to be rapidly reduced. Conclusion: This case highlights that CD can be associated with AD in children, and a gluten-free diet seems to positively influence the course of AD.

## 1. Background

Celiac disease (CD) is an autoimmune disorder that develops because of an aberrant adaptive immune response against gluten-containing grains in genetically predisposed subjects. From a genetic point of view, it can be considered a polygenic disease with a complex pattern of inheritance, involving the major histocompatibility complex (MHC) region and non-MHC genes [1]. However, about 90% of patients with CD carry the alleles encoding HLA-DQ2 and DQ8. Clinically, CD is characterized by a wide spectrum of clinical manifestations, including classic malabsorption with severe diarrhea, extraintestinal features, subclinical and asymptomatic cases, and potential illness. Patients with potential CD have positive serology for antibodies against tissue transglutaminase with normal duodenal biopsy findings [2]. According to the 2012 ESPGHAN guidelines in patients with typical symptoms, anti-tranglutaminase antibodies elevated over 10 times the normal range confirmed by the positivity of anti-endomisium antibodies and by the compatible HLA aplotypes are sufficient to diagnose CD, sparing the intestinal biopsy [3].

Addison’s disease (AD) is a primary adrenal insufficiency, a rare condition with a prevalence of 100 to 140 cases per million inhabitants in Western countries [4]. A short corticotropin test (250 µg) is recommended as the gold standard diagnostic tool for AD. If this test is not immediately possible, an initial screening procedure comprising the measurement of morning plasma adrenocorticotropic hormone (ACTH) and cortisol levels must be considered.

In a number of patients, CD is associated with one or more additional autoimmune diseases, including endocrinopathies [3]. Among these, the most common types are thyroiditis (frequently diagnosed in adults) and type 1 diabetes, which is typical, although not exclusive, of pediatric disease [5]. However, primary AD and CD may co-exist. This has been described particularly in patients with the so-called autoimmune polyglandular syndrome, a rare clinical condition in which a combination of at least two autoimmune endocrinological disorders, including AD, thyroiditis, hypoparathyroidism, type 1 diabetes, or primary gonadal failure, occurs [6]. The combined syndrome of CD with AD in children is relatively uncommon. Moreover, it is not precisely defined whether a gluten-free diet influences the course of AD. A case of CD in a 12-year-old boy presenting as acute adrenal insufficiency is described here.

## 2. Case Presentation

A 12-year-old boy was admitted to the Santa Maria Hospital of Terni on 27 September 2016, due to a three-day history of fever (38 °C) and vomiting with acute presentation. Approximately two weeks before, the child had suffered from an episode of mild fever associated with abdominal pain and diarrhea that spontaneously resolved in a few days. In addition, in the previous three months, the child had complained of fatigue and loss of appetite several times; the previous history was negative. A mild reduction in body weight, of approximately 2 kg, was noted. Finally, in the last year, the parents had noticed mild but significant hyperpigmentation of the skin and mucous membranes, without giving it any importance. The child was previously well, suffering no significant diseases. His physical and neurological development was always considered within the normal range. There was no family history of severe clinical problems, including autoimmune diseases.

**Figure 1:**
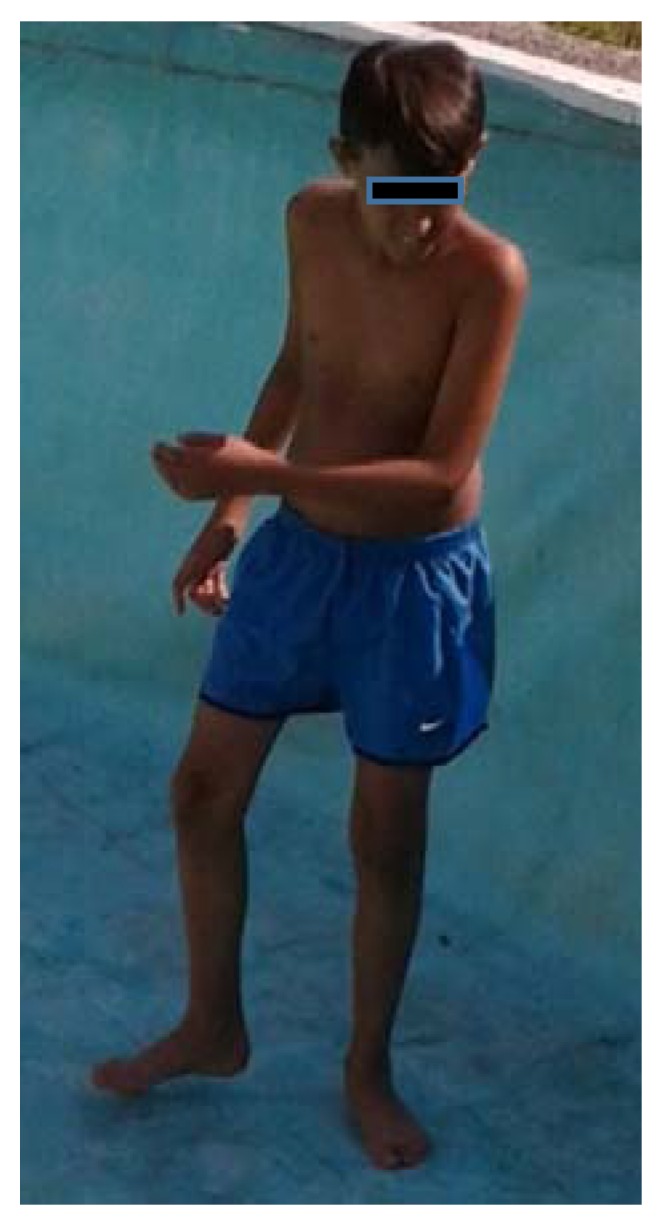
Characteristics of the patient at admission. Physical examination revealed a patient in moderate general condition, with a body weight of 35 kg (28° centile), a height of 150 cm (50° centile), and a body mass index of 15 (10° centile). Marked darkening of the skin, particularly on the palms and areolae, was evident. Subcutaneous fat tissue was reduced.

Figure 1 shows a picture of the boy at the time of admission. Physical examination revealed a patient in mediocre general condition, with a body weight of 35 kg (28° centile), a height of 150 cm (50° centile), and a body mass index of 15 (10° centile). Marked darkening of the skin, particularly on the palms and areolae, was evident. Subcutaneous fat tissue was reduced. No pathological findings of the upper and lower respiratory tract, heart, abdomen, or central or peripheral central nervous system were detected. His blood pressure was 110/60 mmHg, which is within the normal range for his age. Laboratory evaluation revealed no abnormalities of white or red blood cell counts (5320/µL; normal values, 4000–10,000/µL and 4,390,000/µL, respectively), no anemia (Hb 12.8 g/dL; normal values, 12–16 g/dL), no increase in serum concentrations of acute phase reactants (C reactive protein, 0.20 mg/dL; normal values <0.65 mg/dL and procalcitonin <0.05 ng/mL), hyponatraemia (124 mEq/L; normal values, 135–145 mEq/L) and hypochloraemia (93 mEq/L; normal values 100–110 mEq/L), mild hyperkalaemia (5.2 mEq/L; normal values, 3.5–5 mEq/L), borderline hypoglycaemia (69 mg/dL; normal values 70–110 mg/dL), and slight increases in blood urea nitrogen (53 mg/dL, normal values 10–50 mg/dL), serum creatine phosphokinase (909 UI/L; normal values 0–171 UI/L), and serum uric acid (9.5 mg/dL; normal values, 4.8–8.7 mg/dL). His ammonia levels, arterial blood gas, and blood lactate were within the normal range.

Immunological evaluations revealed normal serum IgA (81 mg/dL; normal values, 70–400 mg/dL), IgG (716 mg/dL; normal values, 700–1600 mg/dL), and IgM levels (47 mg/dL; normal values, 40–280 mg/dL), as well as negative anti-nuclear antibodies, anti-neutrophil cytoplasmic antibodies, anti-mitochondrial antibodies, extractable nuclear antigen screening, anti-thyroglobulin antibodies, anti-thyroperoxidase antibodies, and anti-phospholipid antibodies. However, a marked increase in IgA anti-transglutaminase (129 U/mL; normal values, <4 U/mL) and anti-endomysium (positive with a titre of 1/80) antibodies was found. A genetic study with polymerase chain reaction (PCR) DNA amplification and inverse hybridization testing alleles DQA1*05, DQB1*02, and DQB1*03:01 revealed the presence of HLA haplotypes DQ2 and DQ8. Duodenal biopsy could be spared according to the 2012 ESPGHAN CD diagnostic guidelines as anti-tranglutaminase antibodies were elevated over 10 times the normal range (129 UI/mL; normal values, <4 UI/mL), confirmed by the positivity of anti-endomisium antibodies and by the compatible HLA aplotypes.

Beginning with the clinical and laboratory data [7,8], celiac disease was diagnosed. In addition, primary AD was suspected. The diagnosis of CD and AD occurred quite late because the previous history of this child was negative and the parents did not give much importance at the symptoms that occurred in the last year. This was confirmed by low values of morning cortisol (0.8 µg/dL) and dehydroepiandrosterone sulfate (16.2 µg/dL), with adrenocorticotropic hormone (ACTH) concentrations of 1250 pg/mL. No abnormalities in adrenal gland morphology were found in abdominal ultrasonography and magnetic resonance tomography images. The child was treated with intravenous saline solution starting with a bolus of 300 mL over 2 h, followed by a maintenance dose of 1600 mL every 24 h. Serum electrolyte concentrations were evaluated every hour in order to ensure the maintenance of normal values. Normalization of the child’s electrolyte values was established within 3 h and the infusion was discontinued after 48 h.

On the second day of observation, further evaluation of the patient’s adrenal function was planned. The basal aldosterone and renin concentrations were evaluated. The renin level was high (19.10 ng/mL/h), whereas the aldosterone level was low (3.30 pg/mL). The serum ACTH level was lower than the previous day, but higher than the normal values (from >1250 pg/mL on day 1 to 477 pg/mL on day 2; normal values, <46 pg/mL). In addition, ACTH administration only marginally increased the cortisol concentrations at 30 (0.8 µg/dL) and at 60 min (0.6 µg/dL; normal values, 4.3–23.0 µg/dL). A 5-h Synacthen stimulation test confirmed adrenal insufficiency, as serum cortisol values did not reach the minimum normal values at various times of evaluation. The basal cortisol level was undetectable (<50 nmol/L) and failed to rise following Synacthen administration: the 60-min value was <600 nmol/L and the 5-h value was <1000 nmol/L.

Considering the simultaneous co-existence of AD and CD, cortisone acetate was administered at 25 mg at 8 a.m. and 12.5 mg at 4 p.m., and a gluten-free diet was initiated, instructing the patient and his parents on how to follow it correctly. The child was discharged from the hospital after four days, and the follow-up was planned. Thirty days following discharge, the evaluation revealed that the skin hyperpigmentation had almost completely disappeared. The adrenal insufficiency was no longer present (serum sodium, 135 mEq/L, potassium 4.4 mEq/L, chloride 101 mEq/L, glucose 93 mg/dL) and only a small dose of cortisone acetate (37.5 mg/die) was required to maintain normal serum electrolyte and cortisol concentrations. The compliance to the gluten-free diet was confirmed by the patient and his family and was also associated with a significant increase in body weight, of 1.5 kg. The anti-transglutaminase antibodies were dosed at the one-month follow-up control, and were found to still be high (121 UI/mL).

## 3. Discussion

An association between AD and CD has been already reported, although there has been very few recorded pediatric cases. Some small case series have shown that, in subjects suffering from primary AD, the prevalence of CD is higher than in the general population without AD, with values of 5–12% versus approximately 1%, respectively [6]. The relationship between the two diseases is further evidenced by the finding that subjects with CD have a significantly greater risk of developing AD. Elfstrom et al. compared 14,366 CD patients to 70,095 matched controls and found a statistically significantly positive association between CD and subsequent AD (hazard ratio: 11.4; 95% confidence interval: 4.4–29.6) [9]. However, the association is not surprising because CD shares many features with other autoimmune disorders, including primary AD. In all the cases, antibodies against the target tissue and a local inflammatory response with lymphocyte infiltration and cytokine production can be demonstrated. In addition, both diseases have a polygenic mode of inheritance with a strong association, as well as some characteristic genetic backgrounds. The same association with CD has been reported for many other autoimmune diseases. Larizza et al. studied the distribution of HLA-DQ αβ heterodimers in children with type 1 diabetes, autoimmune thyroiditis, and CD alone or associated to other disorders and in 224 healthy controls [10]. It was found that the increased number of HLA-DQ markers of susceptibility for both CD (*p* = 0.006) and type 1 diabetes (*p* = 0.003) was significantly associated with the risk for multiple autoimmune diseases. In addition, the presence of more than one DQ molecules associated with type 1 diabetes significantly increased the risk of developing CD (*p* < 0.001), in addition to diabetes itself and autoimmune thyroiditis (*p* = 0.001). Similarly, the presence of one or more CD HLA-DQ heterodimers significantly increased the likelihood of developing not only CD (*p* < 0.001) but also type 1 diabetes (*p* < 0.001) and autoimmune thyroiditis (*p* < 0.001). As observed in the child described here, the presence of two CD HLA-DQ heterodimers may be associated with the likelihood of having AD.

A gluten-free diet had a significant therapeutic role in this child. Most of the clinical signs and symptoms of AD disappeared in a few days; in the first week, abdominal pain and diarrhea disappeared and the skin pigmentation gradually improved. In addition, the dosage of cortisol acetate, initially administered to face AD, was able to be rapidly reduced. The importance of a gluten-free diet on the incidence and outcome of autoimmune diseases has been largely studied. Unfortunately, data are conflicting. Regarding AD, Betterle at al. reported that dietetic treatment of CD was not effective in avoiding the risk of AD development [11]. Ventura et al. showed that a two-year gluten-free diet was associated with complete normalization of serum levels of autoimmune antibodies previously detected in type 1 diabetes or autoimmune thyroiditis in adults [12]. Similar findings were reported by Cosnes et al., who studied the occurrence of autoimmune diseases and compliance to a gluten-free diet in 924 adults and children with CD [13]. They found that the cumulative risk of subsequent autoimmune diseases was lower in compliant versus non-compliant patients (at 10 years, 6% vs. 15.6%; *p* = 0.02). Age might explain these different results and the positive impact of a gluten-free diet in our patient. Ventura et al. studied 909 patients with CD and found that the prevalence of autoimmune disorders in CD increased with increasing age at diagnosis: 5.1%, 17.1%, and 23.6% in children <2 years old, in those 2–10 years old, and in those >10 years old, respectively (*p* < 0.0001) [14]. In addition, it has been reported that a gluten-free diet improved five patients with this disease and CD [15]. Finally, some data collected in patients with autoimmune thyroiditis seem to indicate that a gluten-free diet can reverse autoimmune disease in childhood but not during adulthood [16]. The case reported here highlights that CD can be associated with AD in children, and a gluten-free diet seems to positively influence the course of AD. Further studies are required to establish whether gluten-free diet could provide some benefit in patients with AD. Considering the rarity of AD, a screening for this condition in patients with CD cannot be recommended, as it would not be cost-effective. However, a high level of suspicion for primary adrenal insufficiency in patients with associated autoimmune conditions, including CD, associated to particular signs and symptoms such as hyponatremia, hypoglycemia, abdominal pain, fever, or hyperpigmentation could reduce the diagnostic delay for AD. In addition, this paper adds to evidence supporting the necessity of screening for CD with anti-transglutaminase antibodies and serum IgA antibodies in subjects with primary adrenal insufficiency.
